# Associations of Sensor-Derived Physical Behavior with Metabolic Health: A Compositional Analysis in the Record Multisensor Study

**DOI:** 10.3390/ijerph16050741

**Published:** 2019-03-01

**Authors:** Isaac Debache, Audrey Bergouignan, Basile Chaix, Emiel M Sneekes, Frédérique Thomas, Cédric Sueur

**Affiliations:** 1 Institut Pluridisciplinaire Hubert Curien (IPHC), UMR 7178 Centre National de la Recherche Scientifique (CNRS), Université de Strasbourg, 67000 Strasbourg, France; audrey.bergouignan@iphc.cnrs.fr (A.B.); cedric.sueur@iphc.cnrs.fr (C.S.); 2 Division of Endocrinology, Metabolism, and Diabetes and Anschutz Health and Wellness Center, School of Medicine, University of Colorado, Aurora, CO 80045, USA; 3 INSERM, Sorbonne Université, Institut Pierre Louis d’Epidémiologie et de Santé Publique, IPLESP, Nemesis team, F75012 Paris, France; basile.chaix@iplesp.upmc.fr; 4 Department of Rehabilitation Medicine, Erasmus MC, 3000 CA Rotterdam, The Netherlands; e.sneekes@erasmusmc.nl; 5 Preventive and Clinical Investigation Center, 75116 Paris, France; Thomas@ipc.asso.fr

**Keywords:** sitting, standing, low physical activity, moderate-to-vigorous physical activity, blood lipids, glucose, HDL, compositional analysis, iso-temporal substitution

## Abstract

Previous studies about the effects of physical activity and sedentary behaviors on health rarely recorded the exact body postures and movements, although they might be of metabolic relevance. Moreover, few studies treated the time budget of behaviors as compositions and little was done to characterize the distribution of durations of behavior sequences in relation with health. Data from the RECORD (Residential Environment and CORonary heart Disease) study of two combined VitaMove accelerometers worn at the trunk and upper leg for a week by 154 male and female adults (age = 50.6 ± 9.6 years, BMI = 25.8 ± 3.9 kg/m^2^) were analyzed. Using both iso-temporal substitution and compositional analysis, we examined associations between five physical behaviors (lying, sitting, standing, low physical activity, moderate-to-vigorous activity) and seven health outcomes (fasting serum glucose, low- and high-density lipoprotein, and triglycerides levels, body mass index, and waist circumference). After adjustment for confounding variables, total standing time was positively associated with better lipid profile, and lying during the day with adiposity. No significant association was observed between breaking up moderate-to-vigorous physical activity and health. This study highlights the importance of refined categories of postures in research on physical activity and health, as well as the necessity for new tools to characterize the distribution of behavior sequence durations, considering both bouts and micro-sequences.

## 1. Introduction

Physical inactivity has been recognized as a major health hazard for several decades [1,2,3]. More recently, research highlighted prolonged sedentary behavior (SB) as a risk factor for developing coronary heart diseases, obesity, diabetes [4,5,6,7], and some cancers [8,9]. This risk factor is thought to operate independently from the level of physical activity (PA) and through different metabolic mechanisms [10,11]. Strictly defined, SB refer to sitting or reclining postures with low energy expenditure (<1.5 metabolic equivalent) [5,12,13]. However, most objective evidence to their adverse effects on health were obtained using a looser definition of SB, based on the sole movement intensity and without distinguishing between quiet standing, sitting and lying. As a consequence, the extent to which the risks associated with SB are distinct from physical inactivity in a narrow sense is still being debated [13,14]. Even among the studies that explicitly distinguished between standing and sitting time, for example with regard to glucose or lipid profile [15,16], only a few investigated the associations between postural behavior and metabolic outcomes in natural, free-living conditions [17,18]. Moreover, the distinction between lying and sitting cannot be properly addressed, even with newer thigh-worn devices such as ActivPal^®^. The study presented here uses a double accelerometer, worn on the subjects’ trunk and thigh, which allows for a precise derivation of body postures and movements.

Independent of the total time spent in MVPA (Moderate to Vigorous Physical Activity) and SB, shorter SB bouts are thought to have a positive effect on cardio-metabolic biomarkers [17,19,20,21]. Yet, although the patterns by which a given SB time is partitioned into sequences of varying durations is relevant for health, most past studies used very simple indices to characterize partitioning patterns, such as the median or the mean bout duration. In the present study, we tested more sophisticated partitioning indices: beside the median bout duration, we used the Gini index of the sequence length distribution [19], and the ratio of behavior time spent in bouts to the total behavior time (spent in bouts or not). By a behavior bout, we mean a sequence of significant duration, e.g., 1 min, during which the dominant observed activity is the activity of interest. Unlike most past studies, we examined the relationship between partitioning patterns and health not only for SB, but also for other behaviors, such as standing or MVPA. In addition, we did not focus exclusively on bouts, but also on sporadic behavior sequences and their distribution.

The associations between the total time volumes spent in each behavior (sitting, standing, MVPA etc.) and a certain health outcome are often estimated in separate models. Whether expressed as time or as proportions, the different behaviors add up to a constant (the total time studied or to 1), and are therefore to be regarded as a time budget with relative and codependent parts: a growing volume of one part always comes at the expense of another. Ignoring this sum constraint often leads to erroneous estimates and interpretations [22,23,24]. Some studies have acknowledged this issue and used iso-temporal substitution techniques [25,26]. These models estimate the effect of time reallocation from one part to another on the outcome variable, while all other parts remain constant. However, others argue that these models still fail at treating these data as proportions, which constitute a sample space, known as the Aitchison simplex, with its own mathematical properties and methods of analysis [24,27,28]. As Biddle and colleagues did in a recent article [18], we address this issue by using both iso-temporal models and methods of compositional analysis.

By using precise categories of physical activities and postures, a thorough approach to duration distribution of activity sequences and appropriate techniques for analyzing time compositions, this study proposes a novel, comprehensive framework for examining the associations between time spent in different physical behaviors, their daily patterns and key health outcomes.

## 2. Material and Methods

### 2.1. Study Subjects

The present study uses data from the MultiSensor sub-study [29] of the RECORD (Residential Environment and CORonary heart Disease) Cohort study. From February 2007 to March 2008, individuals that came to four of the IPC (Investigation Préventive et Clinique) Medical Centers for a free medical examination offered by the French National Insurance System for Employees were invited to enter the RECORD study. Eligibility criteria were age 30–79 years, residence at baseline in 10 districts of Paris (out of 20) and 111 other municipalities of the Ile-de-France region and sufficient cognitive and linguistic abilities. During the second wave of the RECORD Study (September 2013 to June 2015), after completing their medical checkups, participants were systematically invited to enter the RECORD MultiSensor Study whenever monitoring devices were available. In this study, 154 participants (97 men and 57 women), aged 34–83 years, accepted to carry a GPS receiver and the two combined accelerometers placed at the trunk and on the lower limb. The study protocol was approved by the French Data Protection Authority (Decision No. DR-2013-568 on 2/12/2013). All participants signed a written informed consent form.

### 2.2. Anthropological and Biological Data

During the screening visit, participants underwent a medical examination including anthropological measurements and a blood draw in a fasting state. Details about collection of anthropological and biological data can be found elsewhere [30]. Anthropological measurements were made by trained nurses at the medical centers. WC (Waist Circumference) was measured using an inelastic tape placed midway between the lower ribs and the iliac crest, on the mid-axillary line. In this study, we used the following outcomes: serum glucose concentration, plasma triglyceride, high- and low-density lipoprotein (HDL and LDL), as well as body mass index (BMI) and waist circumference (WC).

### 2.3. Physical Activities and Postures

Physical activities and postures were derived from two tri-axial Vitamove Research-V1000^®^ (Vitabase v2.0 B5, Temec Instruments, Herleen, The Netherlands) accelerometers. Participants were requested to wear one at the trunk and the other on the right upper leg during wake time for seven days, as they carried out usual daily activities in free-living conditions (except for water-based activities). In addition, they wore a small GPS device and kept a log of the places they visited and the wearing times of the devices. We invalidated days with less than ten hours of wear time, and subjects with less than four valid days out of seven [31]. Twenty-two subjects out of 154 did not meet these criteria.

The base software of the sensors, VitaScore^®^, has a large nomenclature of physical activities and postures. To simplify the analysis, we combined them into five categories: lying (trunk in horizontal or nearly horizontal position), sitting (trunk upright or nearly upright), standing, light physical activity (LPA, including slow movements) and moderate-to-vigorous physical activity (MVPA, including walking, running, biking etc.).

**Figure 1:**
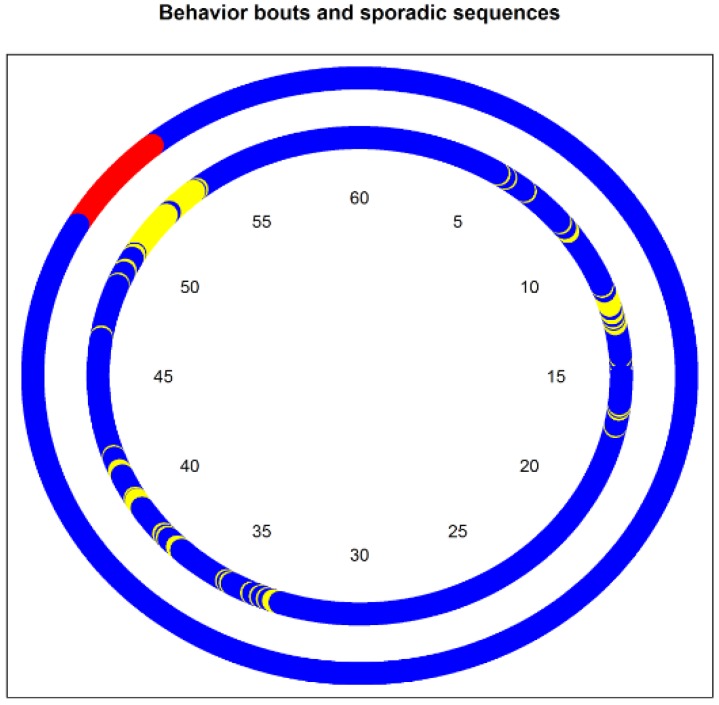
A random sedentary-behavior chart over an hour (based on the empirical distribution of sequence duration). In the inner circle, non-sedentary time is colored in yellow and sedentary time in blue. The outer circle represents, for the same data, the time that is regarded as non-sedentary bout is in red, and as sedentary bouts in blue. This study takes into account both bouts and sporadic sequences, although the latter is disregarded by traditional methodology. Here, 7.6 min were spent in NSB (yellow), and 3.4 in a NSB bout (red). The ratio time in bouts to total time is of 0.42.

To identify bouts, we used a modified version of the function *guideline.bouts* from the R-package *activpalProcessing* [32]. The minimum length of the bouts was set to 60 seconds and the threshold for the proportion of the behavior of interest was kept at 0.8 (see Figure 1). For the sake of simplicity, we did not analyze partitioning patterns for each behavior, but on three broader categories: SB (including lying and sitting), non-sedentary behaviors (NSB) including standing, LPA and MVPA, and MVPA only.

### 2.4. Other Data

Individuals that joined the study answered a questionnaire regarding socio-demographics, dietary and health habits, from which we used data about age, educational level and annual total income. As nutrition is thought to be correlated with both sedentary time and health [33], we also added to the models information regarding nutritional and health habits. These variables are described in details in Table A2 in Appendix C. A discussion about the validity of the questionnaire is to be found in another paper devoted to the RECORD study [34].

### 2.5. Data Processing

BMI and triglycerides data were log transformed in the models. The information about nutrition and health habits was reduced and expressed as the first two dimensions of a principal component analysis performed over the array of all relevant variables in the questionnaire mentioned above. They appear in the tables as “nutritional index”.

### 2.6. Statistical Analysis

The following three models, run with individuals as statistical units, addressed the two questions at hand: the relationship between the behavior time budget and health (the first two models below), and the relationship between the behavior partitioning patterns and health for a given behavior time budget (third model below). In all models, sex, age, annual income, education, and the two nutritional indices were added as control variables. BMI was added as control variable in all the models, except in those whose response variable was BMI or waist circumference.

**Figure 2:**
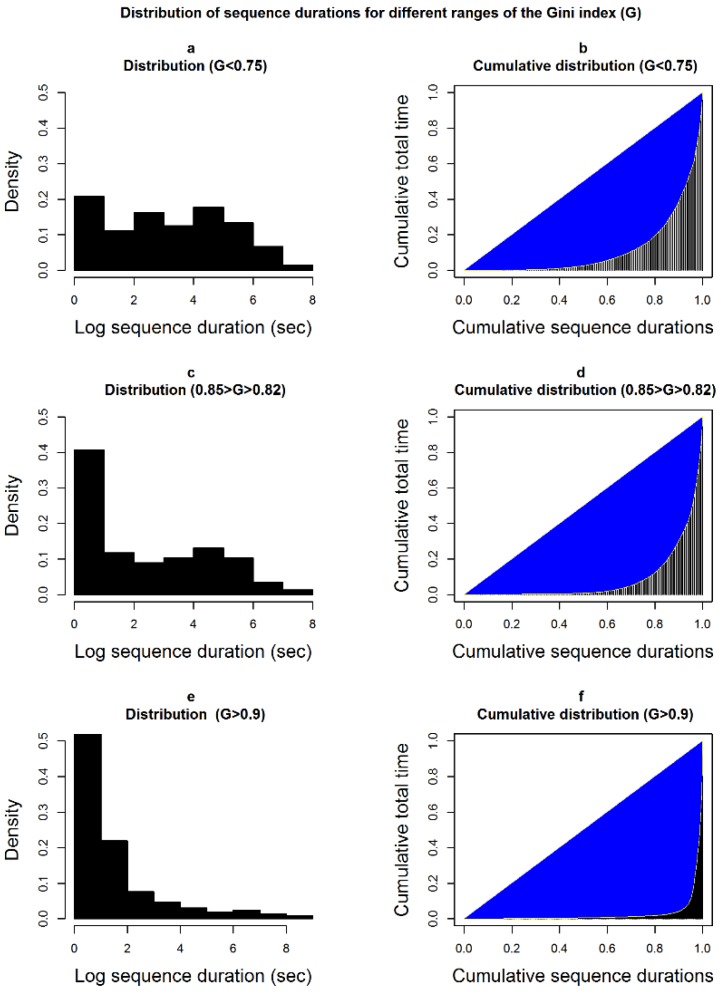
Random non-sedentary sequence durations (sub-figures (**a**,**c**,**e**)) and their corresponding Lorenz curves, i.e., the total time accumulation by sequence duration (sub-figures (**b**,**d**,**f**)), for three different ranges of the Gini index: low (G < 0.75; sub-figures (**a**,**b**)), medium (0.85 > G > 0.82; sub-figures (**c**,**d**)) and high (G > 0.9; sub figures (**e**,**f**)). The durations are randomly drawn from the empirical distributions observed in our population and they add up to the same total time. The Gini index increases as contributions of sequences to the total time are less even (top to bottom). It represents the area between the diagonal and the Lorenz curve (right-hand column) divided by the whole area under the diagonal.

- Iso-temporal substitution models: they estimate the change in the health outcome variable associated with time reallocation (in proportion) from a behavior to another, while all other behavior time volumes remain constant. Thus, the models preserve the compositional structure of the data.
- Compositional models: they are identical to the familiar linear regression models, but before including them as regressors in the models, the compositions are transformed from coordinates in the Aitchison simplex for composition $S^{D}$ to the coordinates in the real space $R^{D-1}$ (here, we chose the isometric log ratio (ilr) transformation [35]). Once the coefficients for the compositions are estimated by the models, they are back-transformed to the Aitchison simplex. The independent variable (here, the health variable) is fitted in the same way as in a traditional linear model, but using the Aitchison geometry for compositions [22] (i.e., by taking the Aitchison inner product of the compositional vector and the corresponding coefficient vector, see Appendix A). Thus, we can estimate our health response variable for any composition, or the change in the response variable following any change in a composition, while operating in the appropriate mathematical framework for these data. To illustrate the change in a health outcome associated with a change in a time budget, we created four hypothetical profiles, which represent archetypes of physical activity patterns, and compared the predicted health outcomes for these profiles against the average profile. The four profiles are ‘couch potato’—a time budget with a strong component of lying/reclining postures (lie = 30%, sit = 50%, stand = 10%, LPA = 5%, MVPA = 5%*)*; ‘office worker’—strong component of sitting (lie = 5%, sit = 70%, stand = 10%, LPA = 5%, MVPA = 10%); ‘doorman’—strong component of standing (lie = 5%, sit = 15%, stand = 70%, LPA = 5%, MVPA = 5%); active—strong component of MVPA (lie = 5%, sit = 40%, stand = 30%, LPA = 5%, MVPA = 20%). We implemented the models using the R-package *compositions* [36] and the handbook by van den Boogaart and Tolosana-Delgado [23].
- Linear models for behavior partitioning: these are traditional linear models, which estimate the change in the health outcome associated with the change in a partitioning index. We did not calculate the indices for each behavior, but rather for three broader categories of behaviors (SB, non-SB, and MVPA). To make sure that the association of behavior partitioning with health is independent of the behavior time volumes, we added the behavior time budget (expressed as ilr) to the model as control variable. As partitioning indices, we use the median length of the behaviors bouts, the ratio of the behavior time in bouts to the total behavior time (spent in bouts or not, see Figure 1), and the Gini index of the total time distribution of sequences of different durations (see Figure 2).

All analyses were performed using the R statistical system (version 3.3.2) [37]. Statistical significance was set at 0.05.

## 3. Results

### 3.1. Anthropological, Demographics and Biological Characteristics of the Participants

The final population was made up of 131 subjects, 64% of them men, aged 50.5 ± 9.6 (arithmetic mean ± standard deviation) years. We removed twenty-two participants for insufficient wear time and one for incomplete biological data. Participants were, in average, slightly overweight with a BMI of 25.8 ± 3.9 kg/m^2^. Seventy-six of the participants were overweight (BMI > 25 kg/m^2^) but only 16 were obese (BMI > 30 kg/m^2^). The others were in normal ranges (20 kg/m^2^ < BMI < 25kg/m^2^). Three participants had metabolic syndrome, as defined by the International Diabetes Foundation [38]. In average, the other health variables examined were in normal ranges. The socio-economic status of the participants was, however, somewhat higher than the French average [39].

### 3.2. Daily Pattern of Physical Activity and Sedentary Behaviors

The mean daily wear time was 14.34 ± 2.08 hours. On average, our population spent 8.04% ± 3.30% of their wake time in MVPA, 3.58% ± 1.40% in LPA, 27.13% ± 9.61% in quiet standing, 51.57% ± 12.08% sitting and 9.68% ± 9.60% lying. Lying time was subject to high inter-individual variability, with values ranging from 0% to 53.63%. The closed geometric mean (which is usually preferred over the arithmetic mean for compositions [23]) was (lie = 5.64%, sit = 54.72%, stand = 27.94%, LPA = 3.64%, MVPA = 8.01%). The covariance matrix, accounting for co-dependencies between the parts of the composition, is shown in Table A1 in Appendix B.

With regard to partitioning patterns, the median bout duration was of 1.8 ± 0.66, 4.37 ± 1.59 and 6.58 ± 2.72 min, for MVPA, NSB and SB, respectively. Although not necessarily related to the median length, the Gini index also points to different partitioning patterns for SB and NSB time than for MVPA, the former being accumulated through fewer, longer sequences (0.8 ± 0.06, 0.83 ± 0.05, and 0.6 ± 0.09, respectively). While the largest share of SB and NSB time was spent in bouts longer than 1 min (0.98 ± 0.02 and 0.96 ± 0.03), the share was much smaller and more variable for MVPA (0.53 ± 0.15).

Detailed descriptive statistics of the physical behaviors and the related indices used in this study are shown in Table 1 and for health and potentially confounding variables in Table 2.

### 3.3. Associations between Behaviors and Health Outcomes

The following section presents the results of our models by health variable. Table 3, Table 4 and Table 5 include full results of the iso-temporal, compositional and partitioning models, respectively. Table 4 also includes the differences (or ratios) in the health values between an average time budget and the four hypothetical time budgets mentioned above.

#### 3.3.1. Blood Glucose Concentration

No significant association was observed between the time volume of any behavior and blood glucose concentration. However, for a given behavioral time budget, partitioning patterns of both SB and NSB time were correlated with glucose level (Table 5). The Gini index was inversely correlated with glucose concentration: glucose level tended to be higher when short and long sedentary sequences contributed to the total time in an equal manner. For example, an increase of 0.1 in the Gini index (see Figure 2) was associated with a decrease of 3.0 mg/dL in glucose concentration. This counter-intuitive result was confirmed by the negative correlation with the ratio (sedentary time in bouts/total sedentary time): as the share of sedentary time spent in bouts decreased, the glucose level increased. A shift from a ratio of 0.9707 (1st quartile) to 0.9892 (3rd quartile) was associated with a decrease of 1.3 mg/dL in glucose level. Although the quadratic term for the ratio was significant, the relation was always negative in the observed range of the ratio values.

NSB (mostly standing) partitioning patterns also correlated with glucose concentration. The relation between the median bout durations of NSB and glucose was U-shaped, with a minimum median length reached at around 6 min. A median bout duration of 3 or 9 min was associated with −3.7 mg/dL and −3.3 mg/dL blood glucose, respectively.

#### 3.3.2. Low-Density Lipoprotein (LDL), High-Density Lipoprotein (HDL), and Triglycerides

Time volumes of MVPA, but also to quiet standing, were clearly associated with triglycerides level, both in the iso-temporal and compositional models (Table 3 and Table 4), and HDL in the iso-temporal model. In this model, reallocation of 1% of the time budget from sitting to standing was associated with an estimated increase in HDL level of 0.2 mg/dL (Table 3). The compositional model for triglycerides concentration confirmed the importance of standing and MVPA (Table 4). The predicted triglycerides concentration for a hypothetical profile ‘doorman’ dominated by standing was 14% lower than that predicted for the average time budget, while the predicted concentration for the profile “active” was 20% lower than for the average profile.

The models including partitioning indices suggest (Table 5) that, independently from the time budget, longer bouts of NSB were associated with lower HDL: an increase of three minutes in the median NSB bout durations was associated with a decrease of 4.8 mg/dL of HDL.

#### 3.3.3. Body Mass Index and Waist Circumference

Only lying time was significantly associated with WC. In the iso-temporal model, reallocating 1% of the total time from lying to sitting, standing, or MVPA was associated with a decrease in WC of 0.34, 0.34 and 0.57 cm, respectively (Table 3). These results are supported by the corresponding compositional model (Table 4). Lying time was clearly associated with higher BMI (reallocating 1% of the total time from lying to standing associated with a decrease of 0.5% in BMI). Interestingly, neither BMI nor WC were associated with MVPA.

For a fixed time budget, the models suggest that longer median NSB bouts are associated with a strong increase in BMI, but not WC: an increase of 3 min in the median length is associated with 6.2% increase in BMI. The model including the Gini index also points to a relationship between NSB partitioning patterns and BMI: as NSB time is accumulated through a smaller number of longer episodes, BMI increases.

## 4. Discussion

Distinguishing between a number of body postures such as standing, sitting and lying, alters our understanding of the relationship between physical activity and health. The beneficial effect of MVPA on the lipid profile has already been well established [40], and we also found positive associations of MVPA time with LDL and triglycerides levels. However, quiet standing, which was often classified as a sedentary behavior [20], was shown here to have similar positive associations with lipid profile (HDL and triglycerides levels). Our models suggest that persons standing during the day (such as the ‘doorman’ in Table 4) have a lower triglycerides level than the average, sedentary individuals. Hence, increasing standing time proportion (e.g., at work) should be studied as a practical alternative to long periods of MVPA. In fact, our finding regarding the importance of standing is supportive of a few other studies, some of which were designed as interventions in the workplace [15,17]. The muscle activation required for posture maintenance, which is more important in standing than sitting [41,42], could explain, at least partly, the beneficial association between standing time and lipid profile. Although both standing time was associated with a better lipid profile, it was not associated with glucose level. This can be explained by the lack of concentric muscle activation in standing, and a relatively low glucose uptake involved with Glucose Transporter type 4 (GLUT-4) translocation [43]. Likewise, the distinction between lying and sitting, which is made possible by the trunk sensor, reveals that lying diverges from sitting in the nature of its associations with BMI and WC in a slightly overweight population. Reallocation of time from lying to all other behaviors, including sitting, was significantly associated with a decrease in WC. This may also be related to a difference in energy expenditure between sitting and lying positions. More generally, the results suggest that, for some health-related aspects, physical activity should be regarded as a gradient in the following order: lying-sitting-standing-MVPA.

Results of the models accounting for the partitioning patterns of behavior time volumes shed light on several associations between physical postures/activities and health. Surprisingly, a certain pattern of partitioning of SB time, namely the existence of a large number of very short sequences of SB beside long sequences (expressed as a high Gini index and a relatively low ratio of bouts to total time) positively associated with fasting plasma glucose concentration. Moreover, glucose concentration was lowest in the population exhibiting a balance between short and long episodes in the accumulation of NSB time. To our knowledge, two studies found a negative correlation between blood glucose concentration and breaking up of SB: Carlson et al. [44] and Bellettiere et al. [17]. The former used a hip-worn accelerometer with the count per minute method, which cannot accurately distinguish between sitting and standing and does not account for sporadic behavior sequences, and the latter did not adjust for total sitting time. In fact, by looking only at bouts of SB, past studies overlooked sporadic sequences of SB, which should be regarded as interruptions in NSB behaviors (i.e., higher levels of physical activity). Many short sequences of SB very likely indicate that the subject does not perform long sequences of NSB, which could easily explain the higher glucose level.

In the same way, the NSB median bout duration was negatively associated with glucose level, up to a certain point. However, the U-shape relationship between NSB median bout duration and glucose suggests that several factors are at play. In fact, we found a positive association between the median duration of NSB bouts and BMI, as well as a negative one with HDL. These results supports the hypotheses proposed by Miles-Chan and Dulloo, according to which it is the efforts associated with frequent transitions between standing and sedentary postures that have a beneficial effect on these variables [41]. These hypotheses might also explain the U-shape observed for glucose level: prolonged standing and PA bouts might be beneficial for health, but shorter bouts points to a higher number of transitions from SB to NSB.

No significant association between health and partitioning patterns of MVPA time. This supports recent similar evidence observed in older British men [45] and is in accordance with the second 2018 US guidelines on physical activity, which removed the recommendation to perform MVPA in bouts of minimum 10 min duration [46]. In other words, any sequence of MVPA matters, regardless of its duration. However, it should be noted that the lack of correlation observed here might be due to the fact that our population did not suffer from severe weight issues.

Overall, compositional models agreed with iso-temporal substitution models. Besides being mathematically appropriate, the former allows a response fitting for any time budget while the latter estimate the response associated with time reallocation from a single part to another. Yet, the interpretation of the compositional models remains less straightforward. In addition, the model provides a significance level for the whole model, but not for each component [24]. We believe that combining both types of models can improve our understanding of the complex relationship between the behavioral time budget and health, and that further elaboration of these procedures of analysis should be a focus of future research.

A main limitation of this study is the impossibility to infer cause-and-effect relationships. In fact, causal links are often counterintuitive. For example, Ekelund et al. showed that it is adiposity that affects the volume of sedentary behaviors, and not inversely [47]. In addition, the relatively small sample size did not allow for investigation of possible effects of interactions with sex or age. Finally, we report results for a healthy population, which might not be extrapolated to other populations, such as those suffering from severe obesity, diabetes or other conditions.

## 5. Conclusions

By distinguishing physical activity and postures, the present study unmasked associations between standing time and lying time with key clinical outcomes, indicating that components other than MVPA play a key role in health. It also showed that the duration of MVPA bouts had no influence on health outcomes. These observations support the newly released U.S. physical activity guidelines that recommend to “Sit Less and Move More” and emphasized the importance of moving all along the day without necessarily trying to attain a specific duration bout of MVPA. Results also suggest that the relationships between fragmentation of SB/NSB and health are more complex than previously assumed and needs to be further investigated. In particular, very short behavior sequences, which have been overlooked in past studies, should be taken into consideration. Future research should focus on innovative ways to link patterns of behavior partitioning to health, use a refined categorization of behaviors and look for ways to implement the new resulting guidelines in the population.

## Figures and Tables

**Table 1:** The top section of the table shows the arithmetic mean, standard deviation (SD), minimum and maximum for the time proportion devoted to each physical behavior (*n* = 131). The bottom sections show the same statistics for various partitioning indices of sedentary time (SB, i.e., lying or sitting), non-sedentary time (NSB, i.e., standing, Light Physical Activity (LPA), Moderate to Vigorous Physical Activity (MVPA)), and MVPA time.

Descriptive Statistics of Physical Activity and Postures	Mean	SD	Min	Max
PHYSCIAL ACTIVTIES & POSTURES (time proportions)				
Lying	0.0968	0.0960	0.0002	0.5363
Sitting	0.5157	0.1208	0.2275	0.7544
Standing	0.2713	0.0961	0.0925	0.6570
LPA	0.0358	0.0140	0.0109	0.0950
MVPA	0.0804	0.0330	0.0168	0.1798
PARTITIONING INDICES (sedentary)				
Median length (minutes)	6.58	2.72	1.73	15.43
Gini	0.7990	0.0608	0.6703	0.9368
Ratio (bouts/total)	0.9770	0.0196	0.8574	0.9958
PARTITIONING INDICES (non-sedentary)				
Median length (minutes)	4.37	1.59	1.52	10.90
Gini	0.8367	0.0502	0.7224	0.9365
Ratio (bouts/total)	0.9613	0.0336	0.7788	0.9964
PARTITIONING INDICES (MVPA)				
Median length (minutes)	1.80	0.66	1.00	5.18
Gini index	0.5954	0.0948	0.3301	0.8159
Ratio (bouts/total)	0.5280	0.1541	0.0797	0.8828

**Table 2:** Arithmetical mean, standard deviation, minimum and maximum values of the health and control co-variables (*n* = 131).

Descriptive Statistics of Health and Control Variables	Mean	SD	Min	Max
Glucose (mg/dL)	96	9	75	123
LDL (mg/dL)	160	38	82	252
HDL (mg/dL)	53	13	25	98
Triglycerides (mg/dL)	109	54	40	306
BMI (kg/m^2^)	25.77	3.89	16.03	37.56
Waist Circumference (cm)	87.34	12.17	57.00	116.00
Sex (0 = female)	0.64			
Age (years)	50.55	9.57	34.00	83.00
Education (categorical)	5.66	2.16	0.00	9.00
Income (categorical)	6.66	2.71	0.00	9.00
Nutritional index 1	0.00	1.55	−3.94	3.08
Nutritional index 2	0.00	1.37	−3.19	4.62

**Table 3:** Estimated coefficient of linear iso-temporal substitution models. The coefficients are the estimated change in *y* due to reallocation of a time unit from one state (column) to another (row). Here, a unit represents the whole time budget. Hence, a reallocation of 1% (0.01) of the total time from sitting to standing, is associated with a change in fasting high density lipoprotein concentration (HDL) of 23.93 × (0.01) ≈ 0.24 mg/dL. Levels of *p*-values: ^†^ <0.1; * <0.05; ** <0.01; *** <0.001.

Results of iso-Temporal Substitution Models (Coefficients and 95% Confidence Intervals)					
Health Outcome	Behavior	Lie	Sit	Stand	LPA
GLUCOSE (mg/dL)	sit	−1.67 [−19.71; 16.37]			
	stand	−9.76 [−33.16; 13.63]	−8.09 [−27.34; 11.15]		
	LPA	61.27 [−81.08; 203.62]	62.94 [−78.69; 204.57]	71.03 [−80.05; 222.12]	
	MVPA	−18.55 [−74.84; 37.74]	−16.88 [−73.26; 39.5]	−8.79 [−66.32; 48.74]	−79.82 [−254.1; 94.45]
LDL (mg/dL)	sit	−60.66 [−134.8; 13.48]			
	stand	−41.59 [−138.16; 54.98]	19.07 [−60.75; 98.89]		
	LPA	136.35 [−453.69; 726.4]	197.02 [−390.29; 784.32]	177.95 [−448.61; 804.5]	
	MVPA	−251.97 * [−484.86; −19.08]	−191.31 [−425.06; 42.45]	−210.38 ^†^ [−448.89; 28.14]	−388.32 [−1111.13; 334.48]
HDL (mg/dL)	sit	−5.07 [−27.36; 17.22]			
	stand	18.87 [−10.17; 47.9]	23.93 * [−0.07; 47.94]		
	LPA	−83.91 [−261.32; 93.5]	−78.84 [−255.43; 97.75]	−102.77 [−291.17; 85.62]	
	MVPA	32.1 [−37.92; 102.13]	37.17 [−33.11; 107.46]	13.24 [−58.48; 84.95]	116.01 [−101.32; 333.34]
log TRIGLYCERIDES (mg/dL)	sit	−0.58 [−1.35; 0.19]			
	stand	−1.33 ** [−2.33; −0.32]	−0.74 ^†^ [−1.58; 0.09]		
	LPA	3 [−3.14; 9.15]	3.59 [−2.53; 9.7]	4.33 [−2.2; 10.86]	
	MVPA	−4 *** [−6.43; −1.57]	−3.42 ** [−5.85; −0.98]	−2.67 * [−5.16; −0.19]	−7 ^†^ [−14.53; 0.53]
log BMI (kg/m^2^)	sit	−0.45 *** [−0.7; −0.2]			
	stand	−0.33 ^†^ [−0.67; 0.01]	0.13 [−0.16; 0.41]		
	LPA	−1.04 [−3.13; 1.05]	−0.59 [−2.68; 1.5]	−0.71 [−2.94; 1.51]	
	MVPA	−0.32 [−1.15; 0.51]	0.13 [−0.7; 0.97]	0.01 [−0.84; 0.86]	0.72 [−1.85; 3.3]
WAIST CIRCUMFERENCE (cm)	sit	−33.53 *** [−49.18; −17.87]			
	stand	−34.28 *** [−55.5; −13.06]	−0.75 [−18.39; 16.88]		
	LPA	−6 [−136.1; 124.11]	27.53 [−102.5; 157.56]	28.28 [−110.33; 166.89]	
	MVPA	−55.69 * [−107.27; −4.1]	−22.16 [−74.12; 29.79]	−21.41 [−74.38; 31.56]	−49.69 [−209.93; 110.54]

**Table 4:** The top section of the table shows the estimated coefficient vectors $\hat{\beta }$ of compositional linear models. If ***x*** is the composition of behavior times, and **z** a vector of co-variables, the predicted outcome *Y* for individual *i* will be: ${\hat{Y}}_{i}=\alpha +{‹\hat{\beta },x_{i}›}_{A}+‹\hat{\gamma },z_{i}›+\epsilon_{i}$. The middle section of the table shows the normalized coefficient vectors, representing the direction along which a composition must be perturbed in order to achieve the largest effect $\left|\hat{\beta }\right|$ on $\hat{Y}$. The bottom section of the table show the change in $\hat{Y}$ associated with four scenarios of departure from the mean composition. The change is expressed as a difference ${\hat{Y}}_{i}-{\hat{Y}}_{M}$ or as ratio $\frac{{\hat{Y}}_{i}}{{\hat{Y}}_{M}}$.

Results of Compositional Models						
COEFFICIENT VECTORS $\hat{\beta }$						
	Glucose	LDL	HDL	Log Trigl.	Log BMI	Waist Circum.
lie	0.3547	0.0198	0.0094	0.209	0.2053	0.748
sit	0.2318	0.0001	0.0008	0.23	0.1948	0.0563
stand	0.0361	0.0051	0.7822	0.1814	0.2002	0.0305
LPA	0.3041	0.975	0.0075	0.2188	0.1968	0.14
MVPA	0.0732	<0.0001	0.2001	0.1608	0.203	0.0253
*p*-value of the model	0.5326	0.2033	0.1858	0.0097	0.0208	0.0006
NORMALIZED COEFFICIENT VECTORS						
lie	0.2854	0.2347	0.1521	0.2183	0.3327	0.3924
sit	0.2305	0.1721	0.0975	0.3025	0.0987	0.1542
stand	0.0906	0.2167	0.3394	0.1347	0.1855	0.1236
LPA	0.2642	0.296	0.1461	0.2551	0.1253	0.2143
MVPA	0.1293	0.0805	0.265	0.0895	0.2578	0.1155
Vector norm $\left|\hat{\beta }\right|$	1.99	16.82	5.51	0.29	0.04	2.77
PREDICTED VALUES compared to mean composition						
Composition (%) [lie, sit, stand, LPA, MVPA]	Diff.	Diff.	Diff.	Ratio	Ratio	Diff.
[30,50,10,5,5]: ‘couch potato’	3.21	13.81	−6.25	1.35	1.03	5.46
[5,70,10,5,10]: ‘office worker’	1.36	−3.67	−4.22	1.12	0.99	0.58
[5,15,70,5,5]: ‘doorman’	−1.35	12.38	6.35	0.86	1.02	0.07
[5,40,30,5,20]: ‘active’	−0.82	−9.79	2.96	0.8	1.01	−1.16

**Table 5:** Coefficients and 95% confidence intervals of linear regression models of various partitioning indices against health variables. The top section refers to sedentary bouts (lying or sitting). The middle section refers to non-sedentary behaviors (standing, Light Physical Activity (LPA) or Moderate to Vigorous Physical Activity (MVPA)). The bottom section refers to MVPA. Quadratic terms are reported when they significantly improve the model. Levels of *p*-values: ^†^ <0.1; * <0.05; ** <0.01; *** <0.001.

Results of Partitioning Models (Coefficients and 95% Confidence Intervals)						
Index	Glucose	LDL	HDL	log Triglycerides	log BMI	Waist Circumf.
SEDENTARY BEHAVIORS						
Median (min.)	−0.41 [−1.1; 0.28]	−0.98 [−3.88; 1.92]	−0.51 [−1.37; 0.36]	0 [−0.03; 0.03]	0 [−0.01; 0.01]	−0.12 [−0.77; 0.53]
Gini	−29.8 * [−56.73; −2.87]	52.19 [−61.82; 166.21]	−6.82 [−40.93; 27.3]	0.13 [−1.07; 1.34]	−0.16 [−0.57; 0.25]	−11.2 [−36.87; 14.46]
Ratio	−3944.26 * [−7423.65; −464.88]	24.26 [−424.35; 472.86]	28.55 [−105.22; 162.33]	0.79 [−3.94; 5.53]	1.45 ^†^ [−0.12; 3.03]	26.26 [−72.96; 125.49]
Ratio^2^	2047.4 * [195.41; 3899.39]					
NON-SEDENTARY BEHAVIORS						
Median (min.)	−4.75 * [−9.31; −0.19]	2.31 [−3.07; 7.68]	−1.61 * [−3.19; −0.02]	0.02 [−0.04; 0.08]	0.02 ** [0.01; 0.04]	0.83 [−0.34; 1.99]
Median^2^ (min.)	0.39 * [0.01; 0.77]					
Gini	13.31 [−20.3; 46.93]	123.44 ^†^ [−15.92; 262.81]	8.68 [−33.42; 50.77]	1.13 [−0.34; 2.61]	0.67 *** [0.21; 1.14]	25.05 ^†^ [−4.61; 54.7]
Ratio	−46.65 [−107.6; 14.3]	−33.56 [−291.17; 224.06]	−63.7 ^†^ [−139.72; 12.31]	0.61 [−2.11; 3.32]	0.28 [−0.64; 1.21]	−2.76 [−60.41; 54.9]
MVPA						
Median (min.)	−0.03 [−2.52; 2.46]	2.29 [−8.12; 12.69]	−0.14 [−3.25; 2.97]	−0.03 [−0.14; 0.08]	−0.01 [−0.05; 0.02]	−1.79 [−4.09; 0.52]
Gini	−1 [−21.87; 19.86]	18.45 [−68.83; 105.73]	9.6 [−16.41; 35.6]	−0.38 [−1.3; 0.54]	−0.12 [−0.44; 0.19]	−8.47 [−27.91; 10.96]
Ratio	−1.09 [−13.77; 11.6]	13.5 [−39.56; 66.57]	3.82 [−12.02; 19.65]	−0.2 [−0.76; 0.35]	−0.05 [−0.24; 0.14]	−2.35 [−14.22; 9.52]

## Appendix A

A few explanations about operations in the Aitchison simplex

- Colsure C:
  $$C\left(a\right)=\frac{a}{1^{t}\cdot a}$$
- Perturbation ⊕:
  $$a⊕b=C\left[a_{1}\cdot b_{1},\ldots ,\;a_{D}\cdot b_{D}\right],$$
  where *D* is the length of the vector.
- Aitchison’s inner product $\langle a,\;{b\rangle }_{A}$:
  $$\langle a,{b\rangle }_{A}=\frac{1}{2D}\;\overset{D}{\underset{i=1}{\sum }}\overset{D}{\underset{j=1}{\sum }}\frac{\ln a_{i}\;}{\ln a_{j}}\;\frac{\ln b_{i}\;}{\ln b_{j}}$$

## Appendix B

Descriptive statistics using a compositional approach

Compositional Mean:

[Lie = 0.05645, Sit = 0.5472, Stand = 0.2794, LPA = 0.0364, MVPA = 0.0805]

Covariance Matrix:

**Table A1:** Covariance matrix of the budget time of lying, sitting, standing, light physical activity and moderate-to-vigorous activity in the population.

Behavior	Lie	Sit	Stand	LPA	MVPA
**Lie**	1.3334	−0.3297	−0.3355	−0.3016	−0.3666
**Sit**	−0.3297	0.2170	0.0380	0.0208	0.0539
**Stand**	−0.3355	0.0380	0.1636	0.0865	0.0475
**LPA**	−0.3016	0.0208	0.0865	0.1265	0.0678
**MVPA**	−0.3666	0.0539	0.0475	0.0678	0.1974

## Appendix C

**Table A2:** Potentially confounding variables controlled for in the models.

Name of Variable	Type of Variable	Value
Education	Continuous	0 = No diploma 1 = Certificat d’études primaire (completion primary school) 2 = Brevet élémentaire ou équivalent (completion of four first year of secondary education) 3 = Certificat d’aptitude professionnelle/Brevet d’études professionnelles (completion of 6 years of secondary vocational education) 4 = Baccalauréat professionnel (completion of vocational secondary cycle) 5 = Baccalauréat general (completion of general secondary cycle) 6 = Bac + 2 (completion of two years of higher education) 7 = Bac + 3 or Bac + 4 (Bachelor’s degree) 8 = Bac + 5 or doctorat (Master’s degree or higher)
Income (Total net revenues of household)	Continuous	0 ≤ 500 € 1 = 500–1000 € 2 = 1000–1500 € 3 = 1500–2000 € 4 = 2000–3000 € 5 = 3000–4000 € 6 = 4000–5000 € 7 = 5000–6000 € 8 = 6000–7000 € 9 ≥ 7000 €
Nutritional habits	The first two dimensions of a principal component analysis including all variables were used as continuous variables.	Intake of olive oil, vegetables, fruits, juice, meat, dairy products, desserts, sodas, wine, legume, fish, pizza, lean meat, nuts, commercial desserts; preference of olive oil over other oils; whether usually eats between meals
